# Integrating radiomics with clinical data for enhanced prediction of vertebral fracture risk

**DOI:** 10.3389/fbioe.2024.1485364

**Published:** 2024-11-22

**Authors:** Babak Saravi, Alisia Zink, Elene Tabukashvili, Hamza Eren Güzel, Sara Ülkümen, Sebastien Couillard-Despres, Gernot Michael Lang, Frank Hassel

**Affiliations:** ^1^ Department of Orthopedics and Trauma Surgery, Faculty of Medicine, Medical Center - University of Freiburg, University of Freiburg, Freiburg, Germany; ^2^ Department of Spine Surgery, Loretto Hospital, Freiburg, Germany; ^3^ Department of Radiology, Ministry of Health Izmir City Hospital, Izmir, Türkiye; ^4^ Institute of Experimental Neuroregeneration, Paracelsus Medical University, Salzburg, Austria; ^5^ Austrian Cluster for Tissue Regeneration, Vienna, Austria

**Keywords:** radiomics, vertebral fractures, CT imaging, machine learning, feature extraction, osteoporosis, artificial intelligence, predictive modeling

## Abstract

**Introduction:**

Osteoporotic vertebral fractures are a major cause of morbidity, disability, and mortality among the elderly. Traditional methods for fracture risk assessment, such as dual-energy X-ray absorptiometry (DXA), may not fully capture the complex factors contributing to fracture risk. This study aims to enhance vertebral fracture risk prediction by integrating radiomics features extracted from computed tomography (CT) scans with clinical data, utilizing advanced machine learning techniques.

**Methods:**

We analyzed CT imaging data and clinical records from 124 patients, extracting a comprehensive set of radiomics features. The dataset included shape, texture, and intensity metrics from segmented vertebrae, alongside clinical variables such as age and DXA T-values. Feature selection was conducted using a Random Forest model, and the predictive performance of multiple machine learning models—Random Forest, Gradient Boosting, Support Vector Machines, and XGBoost—was evaluated. Outcomes included the number of fractures (N_Fx), mean fracture grade, and mean fracture shape. Incorporating radiomics features with clinical data significantly improved predictive accuracy across all outcomes. The XGBoost model demonstrated superior performance, achieving an R^2^ of 0.7620 for N_Fx prediction in the training set and 0.7291 in the validation set. Key radiomics features such as Dependence Entropy, Total Energy, and Surface Volume Ratio showed strong correlations with fracture outcomes. Notably, Dependence Entropy, which reflects the complexity of voxel intensity arrangements, was a critical predictor of fracture severity and number.

**Discussion:**

This study underscores the potential of radiomics as a valuable tool for enhancing fracture risk assessment beyond traditional clinical methods. The integration of radiomics features with clinical data provides a more nuanced understanding of vertebral bone health, facilitating more accurate risk stratification and personalized management in osteoporosis care. Future research should focus on standardizing radiomics methodologies and validating these findings across diverse populations.

## 1 Introduction

Osteoporosis is a common degenerative skeletal condition characterized by low bone mass and the deterioration of bone tissue microarchitecture, leading to increased skeletal fragility and a high risk of fractures (Compston et al., 2019). The prevalence of osteoporotic fractures is expected to rise, potentially comprising 50% of all fractures by 2050 (Compston et al., 2019; Zhu et al., 2023). These fractures are significant contributors to morbidity, mortality, and disability among the elderly (Clynes et al., 2020; Sattui and Saag, 2014). In Europe, the prevalence of osteoporotic vertebral fractures varies between 18% and 26%. In Germany, women are 2.4 times more frequently affected than men (Ballane et al., 2017; Bassgen et al., 2013; Spiegl et al., 2021). The German Osteology Society’s guideline (DVO, Dachverband Osteologie) utilizes the Genant classification system (Genant et al., 1993) to categorize these fractures (Spiegl et al., 2021).

Bone mineral density (BMD) assessment, typically measured by dual-energy X-ray absorptiometry (DEXA), remains the gold standard for osteoporosis diagnosis (Gani et al., 2024). CT-derived Hounsfield units (HU) from vertebral bodies have shown strong correlation with DEXA T-scores and have demonstrated reliability in diagnosing osteoporosis (Pickhardt et al., 2011; Pickhardt et al., 2013). Moreover, advanced imaging techniques have been explored, revealing that bone marrow signal intensity on T1-weighted images is inversely related to osteoporosis, aiding in the development of MRI-based scoring systems for prognostication (Shen et al., 2012; Ehresman et al., 2020). In addition to traditional BMD measurements, subject-specific finite element (FE) models have been developed for solving biomechanically related clinical problems, including bone strength predictions (Molinari and Falcinelli, 2022). These models reconstruct realistic 3-D patient-specific images from radiological data, applying material properties based on Hounsfield units (HU) to FE meshed models. Mechanical, structural, and fracture characteristics are then assessed by applying boundary and loading conditions. Studies have shown that FE-based analysis can reliably predict bone strength (Anitha et al., 2019) and fracture risk (Allaire et al., 2019; Choksi et al., 2018). Volumetric BMD combined with FE-predicted bone strength has been demonstrated to predict fracture risk more accurately than DEXA-based BMD or FE-predicted bone strength alone (Wang et al., 2012). However, despite its potential, FE analysis has not yet become a standard clinical protocol due to concerns over radiation exposure, processing time, and associated costs (Wang et al., 2012).

Radiomics, a burgeoning field, leverages complex algorithms to extract detailed features from medical images, thereby enabling a quantitative evaluation of lesion heterogeneity (Mayerhoefer et al., 2020; Saravi et al., 2023). This approach transforms traditional medical imaging into rich, high-dimensional datasets, facilitating the extraction of features that are not discernible through conventional observation. These radiomic features have shown potential in various musculoskeletal applications, including the assessment of vertebral bone fragility and the evaluation of vertebral load through texture analysis (Zaworski et al., 2021; Sollmann et al., 2021; Tabari et al., 2017; Rastegar et al., 2020; Burian et al., 2019; Valentinitsch et al., 2019). Despite these advancements, there is a scarcity of research specifically focusing on the application of radiomics along with clinical features such as age and DEXA T-scores for predicting vertebral bone fracture risk and fracture characteristics.

Given the critical need for improved fracture risk assessment methods, the current study aims to evaluate the predictive value of radiomics features extracted from CT scans, in conjunction with clinical data such as age and DEXA T-scores, for assessing vertebral fracture risk and fracture characteristics. This study explores the use of machine learning algorithms to identify predictive patterns in a multimodal dataset containing clinical and imaging features (Saravi et al., 2022). The goal is to enhance the understanding of bone health beyond conventional clinical assessments, potentially providing a noninvasive tool for better risk stratification and management in patients at risk of vertebral fractures.

## 2 Methods

### 2.1 Train/test dataset

The dataset utilized for this study was derived from the “MDCT vertebra segmentation and localization dataset”, published as part of the Verse2019 challenge (Löffler et al., 2020). This dataset was collected following approval from the local institutional review board of the Technical University of Munich (proposal 27/19 *S*-SR), with the waiver of written informed consent. The data comprised CT images from two retrospective studies. The inclusion criteria for the first study were the availability of lumbar dual-energy x-ray absorptiometry (DXA) and a CT scan of the lumbar region, both performed within 1 year. For the second study, inclusion required a non-enhanced CT scan of the entire spine. Additional patient selection criteria included being over 30 years of age and having no history of bone metastases.

CT imaging was performed using five different multidetector CT scanners: Philips Brilliance 64, iCT 256, IQon (Philips Medical Care), Siemens Somatom Definition AS, and AS+ (Siemens Healthineers). Scans were executed with a peak tube voltage of 120 kVp, a slice thickness of 0.9–1 mm, and adaptive tube load settings. Some scans included the administration of oral and/or intravenous contrast media. The images were collected in helical mode, and post-contrast scans were taken either in the arterial or portal venous phase.

A total of 104 CT image series from 104 patients were selected based on imaging requirements and the availability of DXA T-values from the dataset. The requirements included acquisition with a 120-kVp peak tube voltage and sagittal reformations reconstructed by filtered back projection favoring sharpness over noise (bone kernel), with a spatial resolution of at least 1 mm in the craniocaudal direction. The scans were obtained between January 2013 and November 2017 and included indications such as acute back pain, suspected spinal fractures, cancer staging, chronic back pain, and postoperative examinations.

Vertebral segmentation was performed in a three-step approach. First, CT data were anonymized and converted to Neuroimaging Informatics Technology Initiative (NIfTI) format, reducing the resolution to limit computational demands. The second step involved the use of a deep learning framework for segmentation, employing a fully convolutional neural network (CNN) to detect the spine, a Btrfly Net for vertebrae labeling on sagittal and coronal maximum intensity projections, and an improved U-Net for segmenting vertebral patches at original resolution. The U-Net was trained on public datasets and continuously retrained with finalized segmentation masks. Finally, the segmentation masks were manually refined by radiologists using ITK-SNAP software (Yushkevich et al., 2016). The segmentation results generated using the 3D Slicer software platform were saved in NIfTI format (.nii.gz) for further processing. The segmentation algorithm used in this study demonstrated a high level of accuracy. It achieved a mean vertebral identification rate of 95.6%, with an average localization error of less than 2 mm. For evaluating the segmentation performance, the Dice similarity coefficient was calculated, yielding a mean score of 91.7% (Liebl et al., 2021).

### 2.2 Validation dataset

For the external validation dataset, we included 20 CT image series from 20 consecutive patients diagnosed with both osteoporotic and non-osteoporotic fractures who underwent CT examinations and dual-energy x-ray absorptiometry (DXA) scans, with available DXA-T values, between January 2020 and May 2023. These patients were recruited from the Department of Spine Surgery at Loretto-Hospital Freiburg, an affiliated hospital of the University Medical Center Freiburg, Germany. Ethical approval was obtained from the local Ethics Committee Freiburg, Germany (Approval Number: 116/200). Informed written consent was secured from all participants before their inclusion in the study.

**FIGURE 1:**
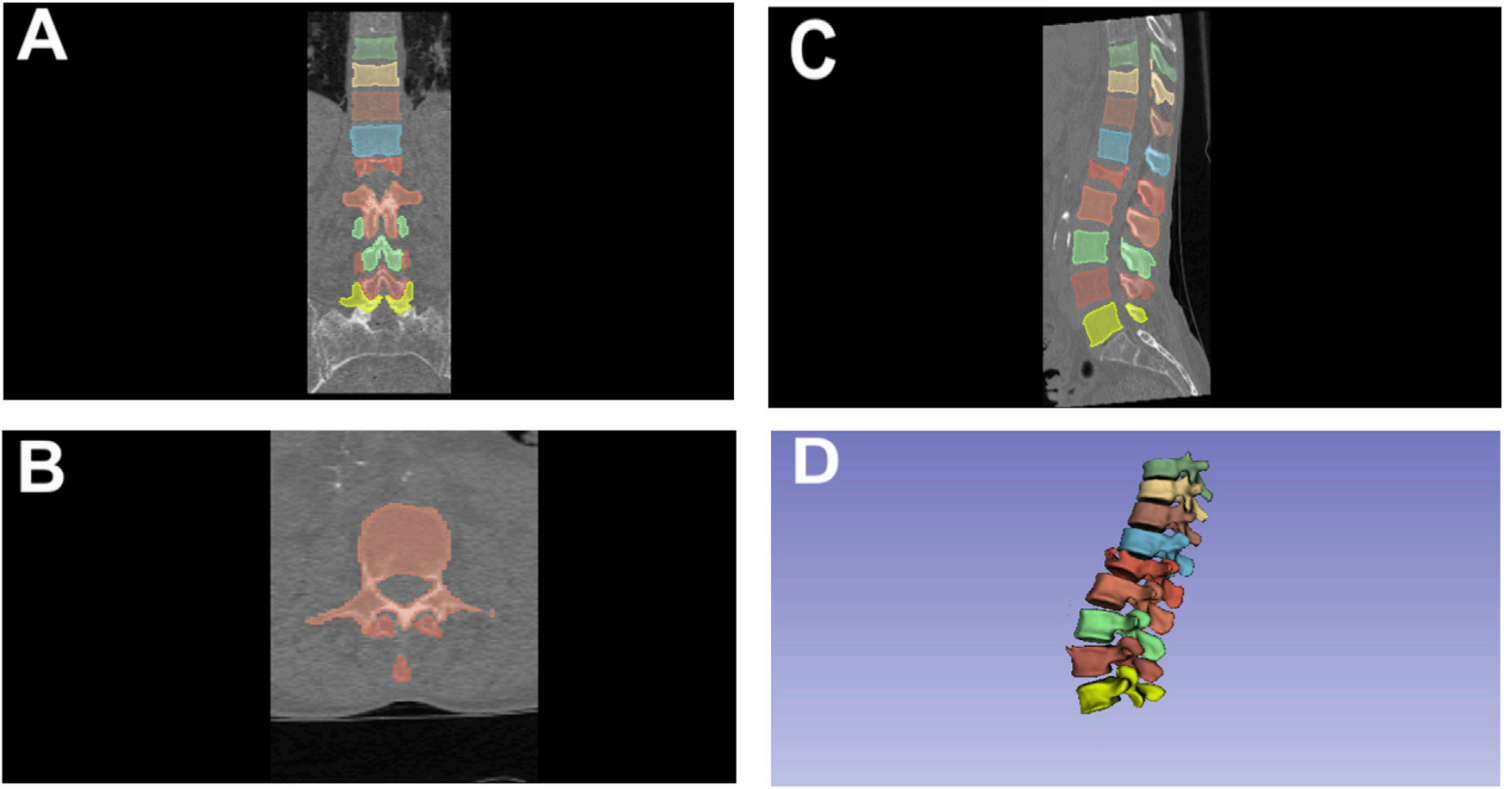
Segmentation of thoracolumbar vertebrae using 3D slicer in various Views. **(A)** Coronal View; **(B)** Axial View; **(C)** Sagittal View; **(D)** 3D rendered view.

CT imaging was performed using one CT scanner (Siemens Somatom go.Up). The segmentation of the vertebrae was carried out by an expert radiologist with over 5 years of experience in image segmentation for artificial intelligence applications. The segmentation process utilized the 3D Slicer software platform (Fedorov et al., 2012), employing the nnU-Net framework (Wasserthal et al., 2023). This framework, based on a U-Net architecture, automatically configures all hyperparameters according to the dataset’s characteristics. The initial segmentations were subsequently refined by the radiologist using the Segment Editor module in 3D Slicer software. An example of the segmentation process is depicted in Figure 1.

### 2.3 Fracture assessment and grading

All CT scans were systematically evaluated for fractures at each thoracolumbar vertebral level, as fractures in the cervical spine are uncommon and typically result from non-osteoporotic causes. The assessment was conducted by a radiologist with over 5 years of experience. Vertebral fractures were classified according to the semiquantitative method established by Genant et al. (1993). This method categorizes fractures based on the percentage of height loss at the vertebral body: fractures were classified as mild with a height loss of ≥20% and <25%, moderate with a height loss of ≥25% and <40%, and severe with a height loss of ≥40%.

Additionally, the type of fracture was determined and categorized into three types: wedge fractures, characterized by the most significant height loss at the anterior aspect of the vertebral body; biconcave fractures, identified by central height loss with nearly equal anterior and posterior height reduction; and crush fractures, marked by the most prominent posterior height loss or uniform reduction including the posterior wall. Vertebral deformities and developmental abnormalities, such as those seen in Scheuermann disease, were excluded from being classified as fractures. The total number of fractures present in the thoracolumbar spine was also documented.

### 2.4 Radiomics feature extraction

Radiomics features were extracted from the segmented vertebrae using the 3D Slicer software platform. The complete list of extracted features is provided in Supplementary Table S1. The features encompass a range of categories, including first-order statistics, shape-based (both 3D and 2D), gray level co-occurrence matrix (GLCM), gray level run length matrix (GLRLM), gray level size zone matrix (GLSZM), neighboring gray-tone difference matrix (NGTDM), and gray level dependence matrix (GLDM). The shape-based features were derived in both 3D and 2D, capturing the geometrical properties of the volume of interest (VOI) without considering the gray level intensity distribution. These features describe aspects such as the size and shape of the VOI, which are calculated solely from the original image and segmentation mask. GLCM features characterize the second-order statistical texture of an image region defined by a mask. They measure the frequency of co-occurrence of pairs of pixel intensities, separated by a specified distance (*δ*) in a given direction (*θ*). Each GLCM matrix element (*i*,*j*) represents the joint probability of pixels having gray levels *i* and *j* at the defined spatial relationship. GLSZM features assess the distribution of homogeneous zones of gray levels within the image. A gray level zone is defined as a group of connected voxels with the same intensity. The GLSZM calculates metrics that quantify the size and intensity variations of these zones. GLRLM features focus on the length of consecutive runs of pixels with the same gray level, capturing the texture patterns in the image based on the uniformity of pixel intensity sequences. NGTDM features provide information on the texture contrast by comparing each pixel’s gray value to the average gray value of its neighboring pixels within a defined distance (*δ*). It calculates the sum of absolute differences for each gray level, offering insights into the local contrast and texture coarseness. GLDM features describe the dependencies among voxels in an image. These features quantify the number of connected voxels within a specific distance (*δ*) that share similar intensity values, thus reflecting the texture’s homogeneity and structure (Mayerhoefer et al., 2020; Parekh and Jacobs, 2016).

### 2.5 Statistical analyses and machine learning algorithms

The clinical features considered were patient age at CT scan (age_ct), sex (sex coded as 0 for female and 1 for male), and the DXA T-value (DXA T-value). Radiomics features, extracted from the segmented vertebral regions, included a range of morphological and textural characteristics (see Supplementary Table S1). Data were standardized using the StandardScaler from scikit-learn to ensure that all features contributed equally to the analysis, without bias due to differing scales. Clinical and radiomics data were combined for comprehensive feature sets used in further analyses. Four machine learning models were selected for evaluation: Random Forest Regressor (RandomForest), Gradient Boosting Regressor (GradientBoosting), Support Vector Regressor (SupportVector), and XGBoost Regressor (XGBoost). These models were chosen due to their proven effectiveness in handling structured data and their ability to capture complex interactions among features. For each model, a pipeline was created comprising the standardization step and the machine learning model itself. This pipeline was essential for ensuring that all transformations were applied consistently across the training and validation datasets.

Hyperparameter optimization was performed using Bayesian Optimization through the BayesSearchCV method from the scikit-optimize library. This method was selected due to its efficiency in exploring the hyperparameter space compared to traditional grid search, particularly for complex models with multiple hyperparameters. Additionally, we implemented class weighting for models to ensure that the minority classes were appropriately represented during training. This approach, along with stratified cross-validation, allowed us to mitigate the impact of class imbalance on model performance and improve generalizability. The optimization process was set to 30 iterations, with a stratified 5-fold cross-validation to evaluate model performance.

The hyperparameters tuned for each model included:

• RandomForest: number of estimators (n_estimators), maximum tree depth (max_depth), minimum samples required to split an internal node (min_samples_split), and minimum samples required at a leaf node (min_samples_leaf).
• GradientBoosting: number of boosting stages (n_estimators), learning rate (learning_rate), maximum tree depth (max_depth), minimum samples to split an internal node (min_samples_split), and minimum samples at a leaf node (min_samples_leaf).
• SupportVector: regularization parameter (C) and the epsilon in the epsilon-SVR model (epsilon).
• XGBoost: number of boosting rounds (n_estimators), maximum tree depth (max_depth), learning rate (learning_rate), subsample ratio (subsample), and column subsample by tree (colsample_bytree).

Radiomics feature selection was performed using a tree-based method, specifically the Random Forest model. The feature importance scores generated by the model were utilized to identify the most predictive features. The Random Forest model’s inherent ability to rank features based on their contribution to the prediction task allowed for an effective selection process. This step was crucial in understanding which features (clinical and radiomics) were most informative in predicting the outcomes of interest: the number of fractures, fracture grading, and fracture shape. Model performance was evaluated using a variety of metrics: Mean Absolute Error (MAE), Mean Squared Error (MSE), Root Mean Square Error (RMSE), and the coefficient of determination (R^2^). These metrics provided a comprehensive assessment of the models’ predictive accuracy, error margins, and overall fit to the data. The correlations were calculated using Pearson or Spearman methods, based on the results of the Shapiro-Wilk normality test. A *p* < 0.05 was considered statistically significant. All analyses were performed in Python.

## 3 Results

### 3.1 Descriptive statistics

The cohort comprised 124 participants, with an average age of 71.46 ± 10.46 years, ranging from 50.6 to 92.7 years (Table 1). The average DXA T-value was −1.61 ± 1.78, spanning from −5.5 to 3.3. On average, 10.04 ± 4.67 thoracolumbar vertebrae were segmented per participant, with a minimum of 4 and a maximum of 17. The mean number of fractures observed was 2.18 ± 2.36, with counts ranging from 0 to 12 fractures.

**TABLE 1:** Descriptive statistics of the cohort.

Variable	Category	Mean ± Std	Min	Max	Count (%)
Age		71.46 ± 10.46	50.6	92.7	
DXA T-value		−1.61 ± 1.78	−5.5	3.3	
Number of Segmented Vertebrae		10.04 ± 4.67	4	17	
Number of Fractures		2.18 ± 2.36	0	12	
Sex	Female				95 (76.61%)
	Male				29 (23.39%)
CT Device	Philips Brilliance 64				49 (39.52%)
	Philips iCT				20 (16.13%)
	Siemens SOMATOM go.Up				20 (16.13%)
	Philips IQon				18 (14.52%)
	Siemens Somatom Definition AS+				17 (13.71%)

The distribution of participants by sex was predominantly female (76.61%, n = 95), with males accounting for 23.39% (n = 29) of the cohort. Various CT devices were used: the Philips Brilliance 64 was the most common (39.52%, n = 49), followed by the Philips iCT and Siemens SOMATOM go.Up (both 16.13%, n = 20), Philips IQon (14.52%, n = 18), and Siemens Somatom Definition AS+ (13.71%, n = 17).

**FIGURE 2:**
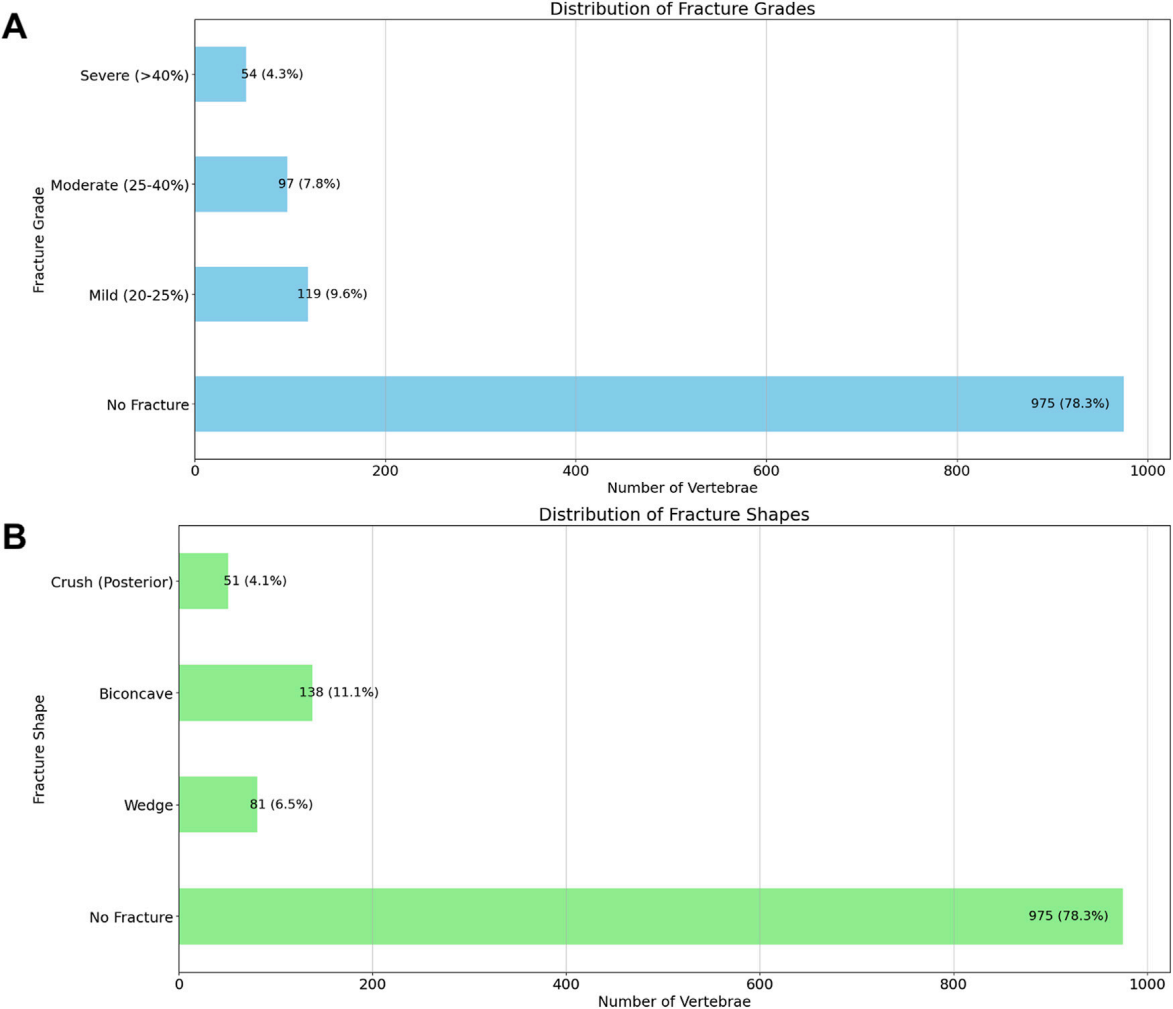
Distribution of Vertebral Fracture Grades **(A)** and Shapes **(B)**. This figure displays the distribution of vertebral fractures based on the Genant classification and fracture shape categories, observed across the segmented vertebrae. The top graph illustrates the distribution of fracture grades, where fractures are classified as No Fracture, Mild (20%–25%), Moderate (25%–40%), or Severe (>40%). The bottom graph depicts the distribution of fracture shapes, categorized as No Fracture, Wedge, Biconcave, or Crush (Posterior). The count and percentage of vertebrae in each category are indicated above each bar.

Regarding vertebral fracture grades, the majority of vertebrae showed no fractures (78.3%, n = 975). Mild fractures (20%–25%) were observed in 9.6% (n = 119) of cases, moderate fractures (25%–40%) in 7.8% (n = 97), and severe fractures (>40%) in 4.3% (n = 54). For fracture shapes, most vertebrae were unfractured (78.3%, n = 975), while 6.5% (n = 81) exhibited wedge fractures, 11.1% (n = 138) showed biconcave fractures, and 4.1% (n = 51) had crush fractures involving the posterior aspect (Figure 2).

### 3.2 Correlation analyses

The correlation analysis identified several significant relationships between the feature variables and the outcomes: mean grade, mean shape, and the number of fractures (N_Fx). For the outcome mean_grade, significant negative correlations were observed with multiple radiomics features, including Flatness (r = −0.362, *p* < 0.0001), 10Percentile (r = −0.426, *p* < 0.0001), Mean (r = −0.403, *p* < 0.0001), and Median (r = −0.404, *p* < 0.0001). A positive correlation was found with DifferenceEntropy (r = 0.200, *p* < 0.05). Additionally, the DXA T-value, a clinical measure of bone density, was significantly negatively correlated with mean_grade (r = −0.346, *p* < 0.0001), indicating that lower bone density is associated with higher fracture grades.

Concerning mean_shape, negative correlations were noted with features such as Flatness (r = −0.348, *p* < 0.0001) and 10Percentile (r = −0.385, *p* < 0.0001). Positive correlations included Imc1 (r = 0.263, *p* < 0.01). The DXA T-value also showed a significant negative correlation with mean_shape (r = −0.339, *p* < 0.001), suggesting that lower bone density correlates with more severe fracture shapes.

**FIGURE 3:**
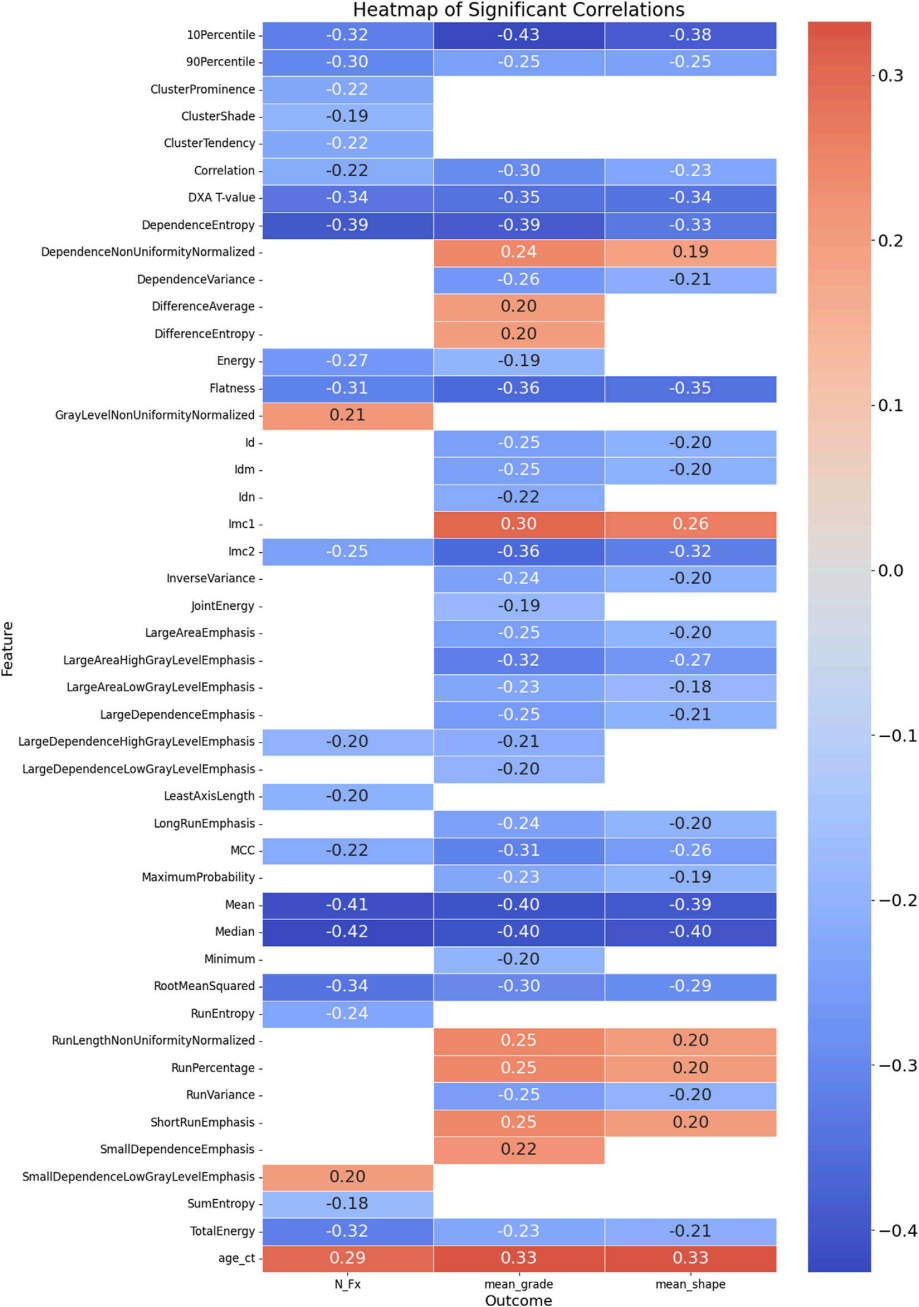
Heatmap of Significant Correlations Between Radiomics Features, DXA T-value, Age, and Fracture Outcomes. This heatmap illustrates the significant correlations between radiomics features, DXA T-value, age at CT examination, and three fracture outcomes: mean grade, mean shape, and number of fractures (N_Fx). The color scale represents the strength and direction of the correlations, with red indicating positive correlations and blue indicating negative correlations. Only the significant correlations (*p* < 0.05) are displayed, providing a visual summary of the relationships between various features and clinical outcomes related to vertebral fractures.

For the outcome N_Fx, significant negative correlations were observed with Intensity-Based Features like Mean (r = −0.410, *p* < 0.0001), Median (r = −0.423, *p* < 0.0001), and RootMeanSquared (r = −0.338, *p* < 0.0001). The DXA T-value had a significant negative correlation with the number of fractures (r = −0.336, *p* < 0.001), emphasizing the relationship between lower bone density and a higher number of fractures. Notably, age was positively correlated with all three outcomes, highlighting its association with increased fracture severity and prevalence. Overall, these findings underscore the utility of radiomics features and DXA T-values in assessing and predicting the severity and type of vertebral fractures. The complete list of significant correlations, along with their correlation coefficients and *p*-values, is detailed in Supplementary Table S2 and Figure 3, providing valuable insights for clinical assessments and potential predictive modeling.

### 3.3 Predictive performance on clinical and clinical + radiomics data

#### 3.3.1 Number of fractures (N_Fx)

We examined the predictive performance of various machine learning models for estimating the number of fractures (N_Fx) using both clinical features alone and a combination of clinical and radiomics features. The models were evaluated on train/test and validation datasets, as summarized in Table 2.

**TABLE 2:** Comparative Performance Metrics of Models for Predicting the Number of Fractures (N_Fx) Using Clinical Features Alone versus Clinical and Top Radiomics Features for the Train/Test and the Validation dataset.

Metrics	Dataset	RandomForest (clinical only)	GradientBoosting (clinical only)	SupportVector (clinical only)	XGBoost (clinical only)	RandomForest (clinical + top radiomics)	GradientBoosting (clinical + top radiomics)	SupportVector (clinical + top radiomics)	XGBoost (clinical + top radiomics)
MAE	Train/Test	1.3984	1.6629	1.6157	1.5076	1.0016	1.5180	1.1172	0.8948
MSE	Train/Test	3.3784	4.8595	5.0606	3.8974	2.0899	4.0453	3.1426	1.4942
RMSE	Train/Test	1.8380	2.2044	2.2496	1.9742	1.4457	2.0113	1.7727	1.2224
R^2^	Train/Test	0.4618	0.2259	0.1939	0.3792	0.6671	0.3556	0.4994	0.7620
MAE	Validation	1.4333	1.7428	1.6792	1.5588	1.0179	1.5427	1.1310	0.9348
MSE	Validation	3.4934	5.0457	5.1154	4.0876	2.1804	4.1201	3.1969	1.5201
RMSE	Validation	1.8787	2.2727	2.3110	2.0169	1.4955	2.0426	1.8111	1.2525
R^2^	Validation	0.4471	0.2217	0.1858	0.3680	0.6496	0.3385	0.4866	0.7291

For models using clinical features alone, the RandomForest model demonstrated a train/test Mean Absolute Error (MAE) of 1.3984 and a Root Mean Square Error (RMSE) of 1.8380. The validation set results were consistent, with only a slight decrease in R^2^ from 0.4618 to 0.4471, indicating a moderate reduction in model performance when applied to unseen data. Similar trends were observed in the GradientBoosting and SupportVector models, which showed less robust fits as indicated by lower R^2^ values compared to RandomForest. The XGBoost model performed relatively better, achieving a train/test R^2^ of 0.3792, but also experienced a drop to 0.3680 in the validation set.

Incorporation of top radiomics features with clinical data significantly enhanced the model performance. Notably, the RandomForest model with combined features achieved a reduced MAE of 1.0016 and an RMSE of 1.4457 on the train/test set, with a corresponding R^2^ improvement to 0.6671. While the validation set exhibited a slight decrease in R^2^ to 0.6496, the performance remained superior compared to clinical-only models.

The XGBoost model, augmented with radiomics features, exhibited the highest accuracy, with an MAE of 0.8948 and an RMSE of 1.2224 in the train/test set, alongside an R^2^ of 0.7620. The validation results showed a minor degradation, with a R^2^ of 0.7291, maintaining the highest predictive power among all evaluated models.

**FIGURE 4:**
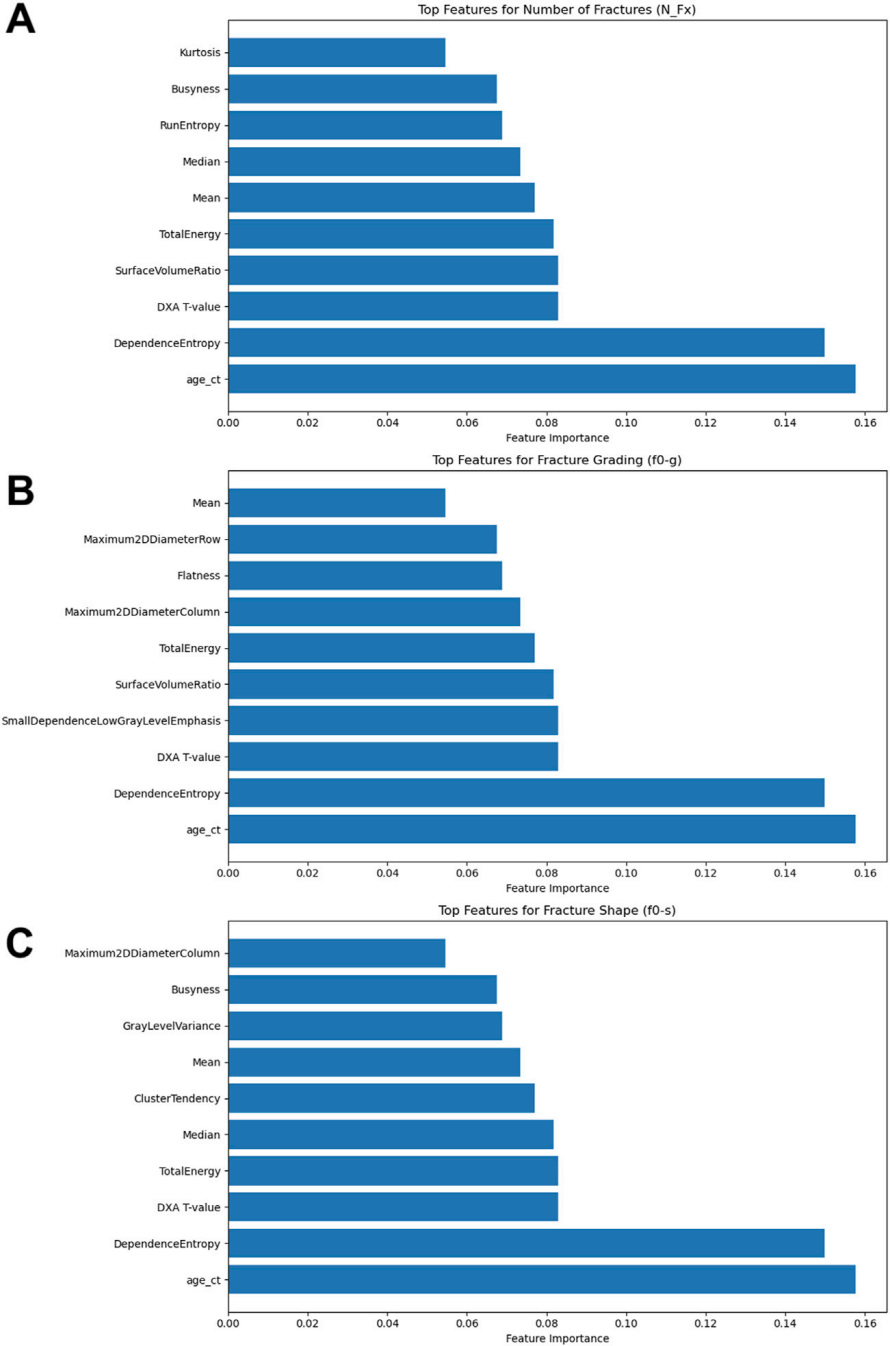
Results of the Feature Importance Analyses. **(A)** Top features for number of fractures (N_Fx); **(B)** Top features for fracture grading (f0-g); **(C)** Top features for fracture shape (f0-s).

The integration of radiomics features provided a notable improvement in the model’s predictive capabilities. The selection of these features was based on their relevance and scores, which highlight their contribution to the model’s decision-making process. The most significant features were as follows (Figure 4):

1. Age at CT (age_ct): This feature had the highest importance score (0.1576), underscoring the critical role of patient age in predicting fracture risk. Age is a well-known clinical determinant in bone health, influencing bone density and susceptibility to fractures.
2. Dependence Entropy: With an importance score of 0.1499, this texture feature reflects the randomness of gray-level intensity distributions in the radiomic analysis. It provides insights into the structural heterogeneity of bone, which can be indicative of bone quality and fracture risk.
3. DXA T-value: This clinical measure, scoring 0.0830, is a key indicator of bone mineral density (BMD) and is commonly used in clinical practice to diagnose osteoporosis. The T-value’s inclusion in the model highlights its relevance in assessing fracture risk.
4. Surface Volume Ratio: This shape feature, with a score of 0.0829, relates to the bone’s surface area relative to its volume, providing information about the geometric complexity of bone structures. A higher surface-to-volume ratio can indicate a higher likelihood of fractures.
5. Total Energy: Scoring 0.0817, this feature measures the sum of the squared elements in the image array, which correlates with the overall signal intensity. It helps in assessing the overall density and texture of the bone, contributing to fracture risk evaluation.
6. Mean: The average intensity value (score 0.0770) represents the central tendency of gray-level intensities, offering a measure of the general radiodensity of the bone.
7. Median: With a score of 0.0735, the median provides a robust measure of central tendency of gray-level intensities, unaffected by outliers, which can be critical in assessing the general state of bone health.
8. Run Entropy: This feature (score 0.0689) quantifies the randomness of the distribution of homogeneous runs in the image. It captures variations in bone texture that could indicate areas of structural weakness.
9. Busyness: Scoring 0.0676, busyness is a texture feature that describes the level of variation in local intensity. High busyness may indicate more complex bone architecture, which can be a factor in fracture risk.
10. Kurtosis: With a score of 0.0546, kurtosis measures the “tailedness” of the intensity distribution, providing insights into the prevalence of extreme values, which can be indicative of anomalies in bone structure.

The integration of these features into predictive models enhanced the accuracy and reliability of fracture risk assessments, as demonstrated by the improved performance metrics across all evaluated models.

#### 3.3.2 Fracture grading (f0-g)

The evaluation of predictive models for fracture grading (f0-g) was conducted using clinical features alone and in combination with top radiomics features. Table 3 presents a comprehensive comparison of these models across both train/test and validation datasets.

**TABLE 3:** Comparative performance metrics of models for predicting fracture grading (f0-g) using clinical features alone versus clinical and top radiomics features.

Metrics	Dataset	RandomForest (clinical only)	GradientBoosting (clinical only)	SupportVector (clinical only)	XGBoost (clinical only)	RandomForest (clinical + radiomics)	GradientBoosting (clinical + radiomics)	SupportVector (clinical + radiomics)	XGBoost (clinical + radiomics)
MAE	Train/Test	0.1401	0.1671	0.1676	0.1417	0.1067	0.1163	0.1442	0.1157
MSE	Train/Test	0.0352	0.0481	0.0507	0.0354	0.0219	0.0230	0.0419	0.0230
RMSE	Train/Test	0.1876	0.2193	0.2252	0.1881	0.1480	0.1517	0.2048	0.1516
R^2^	Train/Test	0.4738	0.2811	0.2419	0.4714	0.6726	0.6558	0.3733	0.6563
MAE	Validation	0.1421	0.1728	0.1702	0.1445	0.1092	0.1215	0.1495	0.1194
MSE	Validation	0.0356	0.0500	0.0520	0.0369	0.0223	0.0233	0.0438	0.0234
RMSE	Validation	0.1925	0.2243	0.2281	0.1963	0.1545	0.1591	0.2100	0.1589
R^2^	Validation	0.4586	0.2746	0.2306	0.4647	0.6470	0.6299	0.3547	0.6377

The RandomForest model using clinical features alone exhibited a Mean Absolute Error (MAE) of 0.1401 and a Root Mean Square Error (RMSE) of 0.1876 on the train/test dataset, with a strong R^2^ value of 0.4738. On the validation set, the model maintained a consistent performance, with slight degradation in R^2^ to 0.4586, indicating a minor reduction in predictive power when applied to new data. The GradientBoosting model, with an MAE of 0.1671 and an RMSE of 0.2193, showed a lower R^2^ of 0.2811 on the train/test set, suggesting less effective performance in capturing variance in fracture grading. The SupportVector model also displayed similar patterns, with metrics closely aligned to GradientBoosting, and a corresponding validation R^2^ decrease to 0.2092. The XGBoost model performed comparably to RandomForest, achieving an R^2^ of 0.4714 on the train/test set and slightly dropping to 0.4647 in the validation set, reinforcing its robustness across datasets.

The inclusion of radiomics features notably improved model performance. The RandomForest model’s MAE decreased to 0.1067, with an RMSE of 0.1480 on the train/test set, and a R^2^ of 0.6726. The validation dataset mirrored these improvements, with an R^2^ of 0.6470, confirming the model’s enhanced predictive capabilities. The GradientBoosting model, similarly augmented with radiomics, achieved an MAE of 0.1163 and an RMSE of 0.1517 on the train/test dataset, with a high R^2^ of 0.6558. Despite a slight reduction, the validation R^2^ remained robust at 0.6299. The SupportVector model, while showing improvement with radiomics features (train/test R^2^ of 0.3733), exhibited a lower R^2^ of 0.3547 on the validation set, highlighting some instability in generalizability. XGBoost demonstrated strong performance, achieving a train/test R^2^ of 0.6563, which only slightly decreased to 0.6377 on the validation set, indicating a highly consistent predictive power across datasets.

The enhanced model performance for fracture grading included key features such as Age at CT (age_ct), Dependence Entropy, DXA T-value, Surface Volume Ratio, Total Energy, Mean, Median, and Busyness (Figure 4). Additionally, unique features such as Small Dependence Low Gray Level Emphasis, which emphasizes small, low-intensity dependencies, provided detailed texture information critical for grading fractures. Flatness, describing the degree of flattening in the bone structure, was also a significant predictor, relevant for identifying specific fracture types. The integration of these radiomics features, alongside clinical data, significantly enhanced the predictive accuracy of the models.

#### 3.3.3 Fracture shape (f0-s)

The assessment of predictive models for fracture shape (f0-s) was conducted using clinical features alone and in combination with top radiomics features. The performance metrics for each model across both train/test and validation datasets are detailed in Table 4.

**TABLE 4:** Comparative performance metrics of models for predicting fracture shape (f0-s) using clinical features alone versus clinical and top radiomics features.

Metrics	Dataset	RandomForest (clinical only)	GradientBoosting (clinical only)	SupportVector (clinical only)	XGBoost (clinical only)	RandomForest (clinical + radiomics)	GradientBoosting (clinical + radiomics)	SupportVector (clinical + radiomics)	XGBoost (clinical + radiomics)
MAE	Train/Test	0.1485	0.1836	0.1769	0.1766	0.1065	0.1115	0.1358	0.0685
MSE	Train/Test	0.0380	0.0548	0.0552	0.0496	0.0214	0.0201	0.0352	0.0087
RMSE	Train/Test	0.1948	0.2340	0.2349	0.2228	0.1464	0.1417	0.1875	0.0934
R^2^	Train/Test	0.4964	0.2733	0.2675	0.3415	0.7157	0.7336	0.5333	0.8842
MAE	Validation	0.1549	0.1862	0.1839	0.1809	0.1091	0.1153	0.1393	0.0703
MSE	Validation	0.0392	0.0569	0.0579	0.0510	0.0220	0.0204	0.0359	0.0091
RMSE	Validation	0.2041	0.2446	0.2433	0.2269	0.1516	0.1439	0.1928	0.0976
R^2^	Validation	0.4767	0.2669	0.2634	0.3265	0.6960	0.7016	0.5103	0.8437

The RandomForest model, utilizing only clinical features, recorded a Mean Absolute Error (MAE) of 0.1485 and a Root Mean Square Error (RMSE) of 0.1948 on the train/test set, with an R^2^ value of 0.4964, indicating a moderate ability to explain variance in fracture shapes. The model’s performance on the validation set showed consistent metrics, with an R^2^ reduction to 0.4767, reflecting a typical decrease in explanatory power when applied to unseen data. The GradientBoosting model achieved an MAE of 0.1836 and an RMSE of 0.2340, with a relatively lower R^2^ of 0.2733 in the train/test set. This performance trend continued into the validation dataset, where the R^2^ decreased to 0.2669. The SupportVector model showed similar performance, with an MAE of 0.1769, an RMSE of 0.2349, and an R^2^ of 0.2675, decreasing to 0.2634 in the validation set. The XGBoost model outperformed the other clinical-only models with an R^2^ of 0.3415 in the train/test set, although it experienced a drop to 0.3265 in the validation set, maintaining the highest accuracy among the clinical-only models.

The introduction of radiomics features resulted in substantial performance improvements. The RandomForest model’s MAE decreased to 0.1065, with an RMSE of 0.1464, and an R^2^ significantly improved to 0.7157 on the train/test set. The validation set exhibited a slight decline in performance, with an R^2^ of 0.6960, indicating robust predictive power. The GradientBoosting model also showed marked improvements, achieving a train/test MAE of 0.1115 and an RMSE of 0.1417. Its R^2^ increased to 0.7336, the highest among the ensemble models. The validation set saw a slight drop in R^2^ to 0.7016, but the model’s performance remained strong. SupportVector models, enhanced with radiomics, showed improvements, with an MAE of 0.1358, RMSE of 0.1875, and R^2^ of 0.5333 on the train/test set. The validation dataset confirmed a decrease in R^2^ to 0.5103, which, while lower, still indicated a notable improvement over clinical-only models. XGBoost, with the addition of radiomics, achieved the best overall performance metrics, with an MAE of 0.0685 and RMSE of 0.0934, along with a R^2^ of 0.8842 on the train/test set. The validation performance, while slightly lower, remained high with an R^2^ of 0.8437, demonstrating the model’s strong generalizability and accuracy.

For predicting fracture shape, the significant features included Age at CT (age_ct), Dependence Entropy, DXA T-value, Total Energy, Median, Busyness, and Kurtosis (Figure 4). Other features like Cluster Tendency, which measures the tendency of voxels with similar gray-level values to form clusters, offered insights into specific bone features and potential weaknesses. Gray Level Variance, indicating variability within the bone structure, was crucial for understanding complex bone shapes. The inclusion of these radiomics features demonstrably enhanced the models’ predictive accuracy for fracture shape prediction.

## 4 Discussion

The primary objective of this study was to evaluate the predictive capability of radiomics features extracted from CT scans, in combination with clinical data, for assessing vertebral fracture risk. We employed machine learning models to identify significant correlations and predict outcomes such as the number of fractures (N_Fx), mean fracture grade, and mean fracture shape. Our analysis revealed that incorporating radiomics features enhances predictive accuracy compared to using clinical data alone.

Several key radiomics features emerged as crucial in the prediction of fracture-related outcomes, such as mean grade, mean shape, and the number of fractures (N_Fx). These features included measures from various categories like texture analysis and shape descriptors, providing a comprehensive assessment of bone quality and structural integrity. Several Texture-Based Features were found to be relevant for prediction modeling. Dependence Entropy, which measures the randomness in the spatial arrangement of voxel intensities, was notably significant across all outcomes, particularly for predicting the number of fractures (N_Fx). This feature captures the complexity of the bone’s internal structure, indicating heterogeneity that might correlate with weaker areas prone to fracture. The high importance score suggests that greater heterogeneity, as indicated by higher entropy, is associated with a greater number of fractures. This insight aligns with the understanding that bones with heterogeneous texture patterns may exhibit compromised structural integrity, increasing fracture risk.

Run Entropy, which quantifies the randomness in the distribution of homogeneous runs within the image, was another critical feature. A higher Run Entropy value reflects more variability in the texture pattern, which could correspond to varying bone quality within a single vertebral segment. This feature was particularly relevant for predicting mean grade and mean shape, indicating that bones with more complex texture patterns may exhibit more severe or varied fracture shapes. Busyness describes the level of local intensity variation and was found to be significant for all outcomes. High busyness may indicate a complex internal bone structure, which could relate to areas of weakness or instability. The feature’s association with fracture outcomes suggests that more varied bone architecture, as reflected by high busyness scores, could be a predictor of increased fracture susceptibility.

Further, Intensity-Based Features were found to be relevant. Both Mean and Median intensity values were negatively correlated with mean grade, mean shape, and the number of fractures. These metrics represent central tendencies of the gray-level intensities within the bone, providing a measure of general radiodensity. Lower values in these features were associated with more severe fractures, indicating that lower bone density, as measured by these intensity metrics, correlates with a higher likelihood and severity of fractures. Total Energy, which measures the sum of squared voxel intensities, reflects the overall signal intensity within the region of interest. This feature was significant in predicting both mean shape and N_Fx, suggesting that lower total energy, indicative of lower overall density, correlates with higher fracture risk. The feature’s importance underscores the utility of total energy as a comprehensive measure of bone density and health.

Moreover, Shape-Based Features were found to be relevant. Surface Volume Ratio, indicating the ratio of the bone’s surface area to its volume, was a significant predictor across multiple outcomes. A higher surface-to-volume ratio can suggest a more complex or irregular bone shape, which may be more prone to fractures. This feature’s relevance highlights the importance of geometric complexity in assessing fracture risk. Further, Flatness and Maximum 2D Diameter were important features. Flatness measures the degree of flattening in the bone structure, while Maximum 2D Diameter assesses the largest span along specific axes. Both features were crucial in determining fracture shape and grading, with higher values potentially indicating more severe deformities. These shape descriptors help in understanding the overall morphology and potential weak points in bone structure.

The incorporation of these radiomics features into predictive models not only improved the models’ accuracy but also provided a more nuanced understanding of the factors contributing to fracture risk. The detailed texture and shape analysis offered by these features could complement traditional clinical measures such as DXA T-values, providing a more comprehensive assessment of bone health. Future research should aim to validate these findings in diverse populations and explore the integration of radiomics into routine clinical workflows. The development of standardized protocols for feature extraction and analysis will be essential to ensure consistency and reproducibility. Furthermore, the potential for radiomics to uncover subtle bone changes not visible through conventional imaging highlights its promise for early intervention and personalized treatment planning.

In recent years, the application of radiomics in the skeletal system has predominantly focused on the evaluation of bone tumors. For instance, Chen et al. (2020) demonstrated that a radiomics nomogram derived from MRI could effectively predict early relapse in osteosarcoma, highlighting the potential of radiomics in oncological prognostication. Similarly, Yin et al. (2019) utilized radiomics features from both CT and MRI to preoperatively differentiate sacral chordomas from giant cell tumors, underscoring the versatility of radiomics in identifying distinct pathological entities. The use of CT radiomics has also been extended to the study of vertebral integrity and structural changes. Muehlematter et al. (2019) illustrated that texture analysis of the spine, combined with support vector machine (SVM) algorithms, could successfully identify vertebrae with fractures. This approach mirrors findings by Li et al. (2023), who reported that CT radiomics, coupled with machine learning, is proficient in detecting occult vertebral fractures that are not easily visible through traditional imaging techniques. These studies collectively suggest that radiomics can enhance the detection and characterization of vertebral pathologies. Further exploring the application of radiomics, Tabari et al. (2017) evaluated trabecular texture analysis in patients with anorexia nervosa, suggesting that specific radiomic parameters may provide valuable insights into bone health, particularly in conditions associated with altered bone metabolism. Complementing these findings, He et al. (2021) proposed that radiomic models, based on lumbar spine MRI, could effectively detect osteoporosis, using both T1-weighted and T2-weighted images to extract relevant features. Additionally, the study by Rastegar et al. (2020) leveraged bone mineral densitometry image features, demonstrating that radiomics combined with machine learning methods can be a novel approach for predicting osteoporosis and osteopenia. This integration of radiomics with traditional bone density measures opens new avenues for non-invasive assessment of bone health. Moreover, radiomics has shown promise in the context of multiple myeloma. One study (Tagliafico et al., 2019) found that radiomics could improve the radiological evaluation of multiple myeloma, distinguishing between focal and diffuse patterns on CT. This capability enhances diagnostic accuracy and could potentially guide treatment decisions. In the realm of vertebral compression fractures, Chee et al. (2021) proposed a combined radiomics-clinical model to predict malignancy, achieving high performance in both training and validation cohorts. The model’s accuracy underscores the potential of radiomics to differentiate between benign and malignant compression fractures, a critical distinction for appropriate clinical management. Furthermore, Yang et al. (2022) demonstrated that a quantitative nomogram incorporating clinical fracture line features and CT radiomic features could distinguish between acute and chronic osteoporotic vertebral fractures with high accuracy. The integration of radiomics features into CT-based finite element (FE) models represents a promising avenue for improving fracture risk prediction. Radiomics offers detailed quantitative insights into bone structure and texture that could complement the biomechanical assessments derived from FE models. By incorporating radiomics features, such as texture complexity and shape descriptors, into FE analysis, it may be possible to achieve a more comprehensive evaluation of bone strength and fracture susceptibility. This combined approach could address current limitations in FE models, such as the assumption of homogeneity in bone material properties, by capturing microstructural variations in bone tissue. Moreover, radiomics could enhance the predictive accuracy of FE models without the need for additional radiation exposure, as it works within the existing CT data framework. While the feasibility of implementing this combined approach in clinical practice would require validation, the potential for more precise, non-invasive fracture risk assessment makes this an innovative direction for future research. While CT-derived Hounsfield units (HU) have traditionally been used to assess bone mineral density and have shown strong correlations with DEXA T-scores, we believe that their addition in our predictive model may offer limited added value. Recent studies have shown that radiomics signatures, which already incorporate intensity-based features such as voxel intensity and texture patterns, provide a more comprehensive assessment of bone morphology alterations. In fact, radiomics models have been demonstrated to outperform HU-based models in predicting bone structure alterations (Jiang et al., 2022). Since the intensity parameters captured by HU are already encompassed within the radiomics features extracted in our study, incorporating HU would likely increase computational complexity and time without a corresponding benefit in predictive accuracy. Therefore, we have opted to focus on radiomics as the primary feature set for enhancing vertebral fracture risk prediction. This study underscores the utility of radiomics in enhancing the temporal characterization of fractures, which is pivotal for determining the appropriate therapeutic approach. To our knowledge, our study is unique in examining the combination of CT radiomics with clinical features to predict not only fracture number but also fracture grading and shape. Further, we focussed on finding the most relevant radiomics features for these tasks. This comprehensive approach represents a significant advancement in the field, potentially providing a more holistic assessment of fracture risk and structural bone integrity. This study contributes to the growing body of evidence supporting the use of radiomics in the evaluation of bone health, particularly in integrating multiple outcomes for a more nuanced understanding of skeletal pathologies.

While the present findings are promising, further research is needed to validate these results across broader populations and diverse imaging settings. Standardizing imaging protocols and feature extraction methods will be critical to ensuring the reproducibility and generalizability of radiomics-based models. Additionally, exploring the integration of other advanced imaging modalities and machine learning techniques could further refine fracture risk prediction. The primary limitations of this study include its retrospective nature, the reliance on a single imaging modality (CT), and the imbalance in the distribution of fracture grades, which could have affected the model’s performance. Although we implemented class weighting and stratified cross-validation to mitigate the impact of this imbalance, future studies may benefit from advanced sampling techniques and larger, more balanced datasets to further improve model robustness. Furthermore, while the machine learning models used in this study offer a comprehensive analysis of clinical and radiomics features, we did not develop a nomogram, which could have provided a more clinically interpretable tool. Future research may explore the creation of nomograms if clinical usability and simpler interpretation are prioritized over the complexity and flexibility offered by machine learning approaches. Future studies should also consider longitudinal designs and the inclusion of additional clinical features such as medication use and comorbidities that could influence bone health. Moreover, one potential area for future research, which has not yet been explored to date, is the combination of FE methods and radiomics for biomechanical analyses and risk stratification.

## 5 Conclusion

This study demonstrates that the integration of radiomics features with clinical data significantly enhances the prediction of vertebral fracture risk. The identified radiomics features, particularly those related to texture and shape, provide valuable quantitative insights that complement traditional clinical assessments. As such, radiomics has the potential to play a crucial role in personalized risk stratification and management of patients at risk of osteoporotic fractures.

To further enhance the practical application of these findings, future research could explore the development of a nomogram that integrates both clinical and radiomics features, providing a more user-friendly tool for clinicians in routine practice. Additionally, the combination of finite element (FE) methods with radiomics represents an exciting and unexplored area of research that could improve biomechanical analyses and fracture risk prediction.

The results underscore the importance of multidisciplinary approaches combining imaging, machine learning, and clinical expertise to advance the field of bone health and osteoporosis research. Future studies should aim to validate these findings in larger, more diverse cohorts and explore the clinical implementation of these advanced imaging biomarkers.

## Data Availability

The raw data supporting the conclusions of this article will be made available by the authors, without undue reservation.
