# Supplementary material for: Integrating radiomics with clinical data for enhanced prediction of vertebral fracture risk
Source: Front Bioeng Biotechnol. 2024 Nov 22;12:1485364. doi: 10.3389/fbioe.2024.1485364 (PMC11620855; doi:10.3389/fbioe.2024.1485364)
Supplement: Supplementary file 1 [file Table1.DOCX]

Supplementary Material

Supplementary Table S1: Radiomics Feature Set.

| Image type | Feature Class | Feature Name |
| --- | --- | --- |
| original | shape | Elongation |
| original | shape | Flatness |
| original | shape | LeastAxisLength |
| original | shape | MajorAxisLength |
| original | shape | Maximum2DDiameterColumn |
| original | shape | Maximum2DDiameterRow |
| original | shape | Maximum2DDiameterSlice |
| original | shape | Maximum3DDiameter |
| original | shape | MeshVolume |
| original | shape | MinorAxisLength |
| original | shape | Sphericity |
| original | shape | SurfaceArea |
| original | shape | SurfaceVolumeRatio |
| original | shape | VoxelVolume |
| original | firstorder | 10Percentile |
| original | firstorder | 90Percentile |
| original | firstorder | Energy |
| original | firstorder | Entropy |
| original | firstorder | InterquartileRange |
| original | firstorder | Kurtosis |
| original | firstorder | Maximum |
| original | firstorder | MeanAbsoluteDeviation |
| original | firstorder | Mean |
| original | firstorder | Median |
| original | firstorder | Minimum |
| original | firstorder | Range |
| original | firstorder | RobustMeanAbsoluteDeviation |
| original | firstorder | RootMeanSquared |
| original | firstorder | Skewness |
| original | firstorder | TotalEnergy |
| original | firstorder | Uniformity |
| original | firstorder | Variance |
| original | glcm | Autocorrelation |
| original | glcm | ClusterProminence |
| original | glcm | ClusterShade |
| original | glcm | ClusterTendency |
| original | glcm | Contrast |
| original | glcm | Correlation |
| original | glcm | DifferenceAverage |
| original | glcm | DifferenceEntropy |
| original | glcm | DifferenceVariance |
| original | glcm | Id |
| original | glcm | Idm |
| original | glcm | Idmn |
| original | glcm | Idn |
| original | glcm | Imc1 |
| original | glcm | Imc2 |
| original | glcm | InverseVariance |
| original | glcm | JointAverage |
| original | glcm | JointEnergy |
| original | glcm | JointEntropy |
| original | glcm | MCC |
| original | glcm | MaximumProbability |
| original | glcm | SumAverage |
| original | glcm | SumEntropy |
| original | glcm | SumSquares |
| original | gldm | DependenceEntropy |
| original | gldm | DependenceNonUniformity |
| original | gldm | DependenceNonUniformityNormalized |
| original | gldm | DependenceVariance |
| original | gldm | GrayLevelNonUniformity |
| original | gldm | GrayLevelVariance |
| original | gldm | HighGrayLevelEmphasis |
| original | gldm | LargeDependenceEmphasis |
| original | gldm | LargeDependenceHighGrayLevelEmphasis |
| original | gldm | LargeDependenceLowGrayLevelEmphasis |
| original | gldm | LowGrayLevelEmphasis |
| original | gldm | SmallDependenceEmphasis |
| original | gldm | SmallDependenceHighGrayLevelEmphasis |
| original | gldm | SmallDependenceLowGrayLevelEmphasis |
| original | glrlm | GrayLevelNonUniformity |
| original | glrlm | GrayLevelNonUniformityNormalized |
| original | glrlm | GrayLevelVariance |
| original | glrlm | HighGrayLevelRunEmphasis |
| original | glrlm | LongRunEmphasis |
| original | glrlm | LongRunHighGrayLevelEmphasis |
| original | glrlm | LongRunLowGrayLevelEmphasis |
| original | glrlm | LowGrayLevelRunEmphasis |
| original | glrlm | RunEntropy |
| original | glrlm | RunLengthNonUniformity |
| original | glrlm | RunLengthNonUniformityNormalized |
| original | glrlm | RunPercentage |
| original | glrlm | RunVariance |
| original | glrlm | ShortRunEmphasis |
| original | glrlm | ShortRunHighGrayLevelEmphasis |
| original | glrlm | ShortRunLowGrayLevelEmphasis |
| original | glszm | GrayLevelNonUniformity |
| original | glszm | GrayLevelNonUniformityNormalized |
| original | glszm | GrayLevelVariance |
| original | glszm | HighGrayLevelZoneEmphasis |
| original | glszm | LargeAreaEmphasis |
| original | glszm | LargeAreaHighGrayLevelEmphasis |
| original | glszm | LargeAreaLowGrayLevelEmphasis |
| original | glszm | LowGrayLevelZoneEmphasis |
| original | glszm | SizeZoneNonUniformity |
| original | glszm | SizeZoneNonUniformityNormalized |
| original | glszm | SmallAreaEmphasis |
| original | glszm | SmallAreaHighGrayLevelEmphasis |
| original | glszm | SmallAreaLowGrayLevelEmphasis |
| original | glszm | ZoneEntropy |
| original | glszm | ZonePercentage |
| original | glszm | ZoneVariance |
| original | ngtdm | Busyness |
| original | ngtdm | Coarseness |
| original | ngtdm | Complexity |
| original | ngtdm | Contrast |
| original | ngtdm | Strength |

Supplementary Table S2: Significant correlations between between radiomics features, DXA T-value, age at CT examination, and three fracture outcomes: mean grade, mean shape, and number of fractures (N_Fx).

| **Feature** | **Outcome** | **Correlation** | **P-value** |
| --- | --- | --- | --- |
| Flatness | mean_grade | -0,36155 | 3,69E-05 |
| 10Percentile | mean_grade | -0,42581 | 8,21E-07 |
| 90Percentile | mean_grade | -0,24791 | 0,005502 |
| Energy | mean_grade | -0,19029 | 0,034272 |
| Mean | mean_grade | -0,40329 | 3,41E-06 |
| Median | mean_grade | -0,40352 | 3,37E-06 |
| Minimum | mean_grade | -0,19507 | 0,029923 |
| RootMeanSquared | mean_grade | -0,29618 | 0,000838 |
| TotalEnergy | mean_grade | -0,22807 | 0,010846 |
| Correlation | mean_grade | -0,29546 | 0,000864 |
| DifferenceAverage | mean_grade | 0,199852 | 0,026053 |
| DifferenceEntropy | mean_grade | 0,197348 | 0,028022 |
| Id | mean_grade | -0,24607 | 0,005871 |
| Idm | mean_grade | -0,24694 | 0,005694 |
| Idn | mean_grade | -0,22103 | 0,013626 |
| Imc1 | mean_grade | 0,304746 | 0,000579 |
| Imc2 | mean_grade | -0,36333 | 3,36E-05 |
| InverseVariance | mean_grade | -0,24258 | 0,006635 |
| JointEnergy | mean_grade | -0,18549 | 0,039154 |
| MCC | mean_grade | -0,31259 | 0,000408 |
| MaximumProbability | mean_grade | -0,22963 | 0,010301 |
| DependenceEntropy | mean_grade | -0,38883 | 8,09E-06 |
| DependenceNonUniformityNormalized | mean_grade | 0,241875 | 0,006801 |
| DependenceVariance | mean_grade | -0,26345 | 0,003113 |
| LargeDependenceEmphasis | mean_grade | -0,25466 | 0,004314 |
| LargeDependenceHighGrayLevelEmphasis | mean_grade | -0,21113 | 0,018582 |
| LargeDependenceLowGrayLevelEmphasis | mean_grade | -0,20121 | 0,025032 |
| SmallDependenceEmphasis | mean_grade | 0,221245 | 0,013534 |
| LongRunEmphasis | mean_grade | -0,24271 | 0,006607 |
| RunLengthNonUniformityNormalized | mean_grade | 0,247375 | 0,005607 |
| RunPercentage | mean_grade | 0,246253 | 0,005834 |
| RunVariance | mean_grade | -0,24949 | 0,005199 |
| ShortRunEmphasis | mean_grade | 0,245468 | 0,005998 |
| LargeAreaEmphasis | mean_grade | -0,24603 | 0,00588 |
| LargeAreaHighGrayLevelEmphasis | mean_grade | -0,31756 | 0,000326 |
| LargeAreaLowGrayLevelEmphasis | mean_grade | -0,23238 | 0,009402 |
| age | mean_grade | 0,330793 | 0,000175 |
| DXA_T_value | mean_grade | -0,34592 | 8,31E-05 |
| Flatness | mean_shape | -0,34768 | 7,6E-05 |
| 10Percentile | mean_shape | -0,38478 | 1,02E-05 |
| 90Percentile | mean_shape | -0,24501 | 0,006094 |
| Mean | mean_shape | -0,39115 | 7,06E-06 |
| Median | mean_shape | -0,40208 | 3,67E-06 |
| RootMeanSquared | mean_shape | -0,29201 | 0,000999 |
| TotalEnergy | mean_shape | -0,21125 | 0,018512 |
| Correlation | mean_shape | -0,23419 | 0,008848 |
| Id | mean_shape | -0,20387 | 0,023138 |
| Idm | mean_shape | -0,20433 | 0,022828 |
| Imc1 | mean_shape | 0,262613 | 0,003213 |
| Imc2 | mean_shape | -0,31927 | 0,000301 |
| InverseVariance | mean_shape | -0,20485 | 0,022473 |
| MCC | mean_shape | -0,2567 | 0,004003 |
| MaximumProbability | mean_shape | -0,19429 | 0,030596 |
| DependenceEntropy | mean_shape | -0,33366 | 0,000153 |
| DependenceNonUniformityNormalized | mean_shape | 0,192255 | 0,032421 |
| DependenceVariance | mean_shape | -0,214 | 0,017007 |
| LargeDependenceEmphasis | mean_shape | -0,20645 | 0,021418 |
| LongRunEmphasis | mean_shape | -0,19524 | 0,029778 |
| RunLengthNonUniformityNormalized | mean_shape | 0,203131 | 0,023654 |
| RunPercentage | mean_shape | 0,199497 | 0,026325 |
| RunVariance | mean_shape | -0,2017 | 0,024677 |
| ShortRunEmphasis | mean_shape | 0,20167 | 0,024698 |
| LargeAreaEmphasis | mean_shape | -0,19552 | 0,029541 |
| LargeAreaHighGrayLevelEmphasis | mean_shape | -0,26649 | 0,002774 |
| LargeAreaLowGrayLevelEmphasis | mean_shape | -0,18472 | 0,039997 |
| age | mean_shape | 0,332344 | 0,000163 |
| DXA_T_value | mean_shape | -0,33895 | 0,000118 |
| Flatness | N_Fx | -0,31331 | 0,000395 |
| LeastAxisLength | N_Fx | -0,20476 | 0,022531 |
| 10Percentile | N_Fx | -0,31631 | 0,000345 |
| 90Percentile | N_Fx | -0,29929 | 0,000734 |
| Energy | N_Fx | -0,2656 | 0,00287 |
| Mean | N_Fx | -0,40977 | 2,29E-06 |
| Median | N_Fx | -0,42317 | 9,75E-07 |
| RootMeanSquared | N_Fx | -0,33829 | 0,000122 |
| TotalEnergy | N_Fx | -0,31632 | 0,000345 |
| ClusterProminence | N_Fx | -0,2248 | 0,01207 |
| ClusterShade | N_Fx | -0,19262 | 0,032089 |
| ClusterTendency | N_Fx | -0,2247 | 0,012109 |
| Correlation | N_Fx | -0,21787 | 0,015064 |
| Imc2 | N_Fx | -0,24676 | 0,00573 |
| MCC | N_Fx | -0,21946 | 0,014326 |
| SumEntropy | N_Fx | -0,18198 | 0,043085 |
| DependenceEntropy | N_Fx | -0,39476 | 5,7E-06 |
| LargeDependenceHighGrayLevelEmphasis | N_Fx | -0,2012 | 0,025042 |
| SmallDependenceLowGrayLevelEmphasis | N_Fx | 0,200857 | 0,025296 |
| GrayLevelNonUniformityNormalized | N_Fx | 0,208593 | 0,020077 |
| RunEntropy | N_Fx | -0,23619 | 0,008266 |
| age | N_Fx | 0,294072 | 0,000916 |
| DXA_T_value | N_Fx | -0,33622 | 0,000135 |
